# Response of physiological parameters in *Dionaea muscipula* J. Ellis teratomas transformed with *rol*B oncogene

**DOI:** 10.1186/s12870-021-03320-y

**Published:** 2021-11-29

**Authors:** Wojciech Makowski, Aleksandra Królicka, Barbara Tokarz, Karolina Miernicka, Anna Kołton, Łukasz Pięta, Kamilla Malek, Halina Ekiert, Agnieszka Szopa, Krzysztof Michał Tokarz

**Affiliations:** 1 Department of Botany, Physiology and Plant Protection, Faculty of Biotechnology and Horticulture, University of Agriculture in Krakow, Krakow, Poland; 2 University of Gdansk, Intercollegiate Faculty of Biotechnology UG and MUG, Laboratory of Biologically Active Compounds, Gdansk, Poland; 3 Jagiellonian University in Krakow, Faculty of Chemistry, Krakow, Poland; 4 Department of Pharmaceutical Botany, Jagiellonian University, Medical College, Krakow, Poland

**Keywords:** *Rhizobium rhizogenes*, Primary and secondary metabolism, Venus flytrap, *Rol* genes, Transformation

## Abstract

**Background:**

Plant transformation with *rol* oncogenes derived from wild strains of *Rhizobium rhizogenes* is a popular biotechnology tool. Transformation effects depend on the type of *rol* gene, expression level, and the number of gene copies incorporated into the plant’s genomic DNA. Although *rol* oncogenes are known as inducers of plant secondary metabolism, little is known about the physiological response of plants subjected to transformation.

**Results:**

In this study, the physiological consequences of *rol*B oncogene incorporation into the DNA of *Dionaea muscipula* J. Ellis was evaluated at the level of primary and secondary metabolism. Examination of the teratoma (transformed shoots) cultures of two different clones (K and L) showed two different strategies for dealing with the presence of the *rol*B gene. Clone K showed an increased ratio of free fatty acids to lipids, superoxide dismutase activity, synthesis of the oxidised form of glutathione, and total pool of glutathione and carotenoids, in comparison to non-transformed plants (control). Clone L was characterised by increased accumulation of malondialdehyde, proline, activity of superoxide dismutase and catalase, total pool of glutathione, ratio of reduced form of glutathione to oxidised form, and accumulation of selected phenolic acids. Moreover, clone L had an enhanced ratio of total triglycerides to lipids and accumulated saccharose, fructose, glucose, and tyrosine.

**Conclusions:**

This study showed that plant transformation with the *rol*B oncogene derived from *R. rhizogenes* induces a pleiotropic effect in plant tissue after transformation. Examination of *D. muscipula* plant in the context of transformation with wild strains of *R. rhizogenes* can be a new source of knowledge about primary and secondary metabolites in transgenic organisms.

**Supplementary Information:**

The online version contains supplementary material available at 10.1186/s12870-021-03320-y.

## Background

Plant transformation with wild strains of *Rhizobium rhizogenes* (former: *Agrobacterium rhizogenes*) bacteria has been a popular tool used in biotechnology for decades [1]. This method is based on the natural ability of *R. rhizogenes* to pass a fragment of the Ri (root-inducing) plasmid and incorporate T-DNA (transfer DNA) into host genomic DNA [2]. Such an event leads to the formation of tumours, hairy roots, or teratomas (transformed shoots) because the expression of bacterial genes in the plant genome disturbs auxin and cytokinin synthesis pathways, which affects the hormonal balance in plant tissue [3]. Transformed plants are usually characterised by fast growth and long-term genetic and biochemical stability, which makes them a good model in the research and industrial field [2]. In terms of the transformation effects, the most important are oncogenes belonging to the *rol* family (*rol*A, *rol*B, *rol*C, *rol*D), which are part of bacterial T-DNA. Although *rol* genes have been studied for many years, clear knowledge of the complete mechanism of how these genes work is missing [1]. Until now, a few reports have shown possible scenarios of the action of *rol* genes in plant cells [4–8]. However, the whole picture is still missing.

One of the most popular and useful oncogenes in plant transformation is *rol*B [9]. This gene is the most powerful inducer of plant secondary metabolism, which is why *rol*B-transformed plants are models in the medical plant area, as well as in research concentrated on plant secondary metabolism [3]. Expression of the *rol*B oncogene can increase the level of secondary metabolites in plant tissue [10, 11], suppress reactive oxygen species (ROS) production [4], and modulate the antioxidant defence system [8]. Moreover, Veremeichik et al. [12] showed that *rol*B expression regulates the activity of NADPH oxidase, while Bulgakov et al. [5] reported the tyrosine phosphatase activity of the rolB protein. Nevertheless, the complex physiological response of medical plants transformed with the *rol*B oncogene has never been studied. To the best of our knowledge, this article is the first report about the response of carnivorous plants to the expression of the *rol*B gene studied primarily at the level of primary metabolism, and consequently secondary metabolism.

*Dionaea muscipula* J. Ellis (Venus flytrap), belonging to the Droseraceae family, is an interesting model for research in plant physiology and secondary metabolite production. This unique carnivorous plant is known as a rich source of phenolic compounds, particularly 1,4-naphthoquinone [13]. Because of a huge demand for plant material with a high concentration of phenolic compounds [14], plants from the Droseraceae family have become an important research model in modern plant biotechnology [15]. *D. muscipula* tissue has a strong biologically active potential [16, 17]. Additionally, Makowski et al. [11] reported the first genetic transformation of the Venus flytrap with wild strains of *R. rhizogenes* bacteria.

**Fig. 1:**
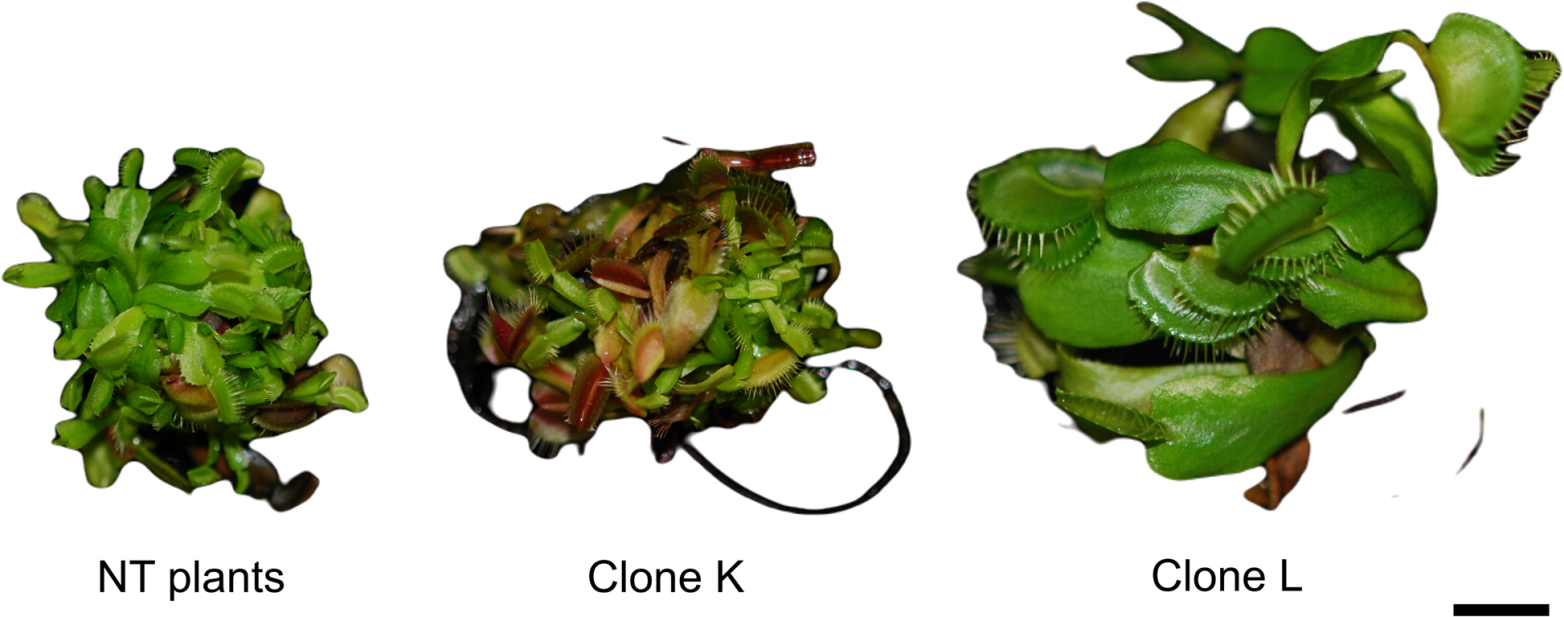
Non-transformed (NT plants) and transformed clones (clones K and L) of *Dionaea muscipula* plants. Scale bar = 1 cm

In the present study, we examined two clones (teratomas) of *D. muscipula* (clones K and L) selected from our previous research. At the molecular level, both plants were transformed with the *rol*B gene, and it was incorporated into plant genomic DNA in a single copy [11]. Independent of the *R. rhizogenes* strain, such transformation types led to the creation of teratoma (transformed shoots) cultures (Fig. 1). Nevertheless, the results of this research showed that plants differed from each other in terms of growth rate, accumulation of dry matter, and phenolic compound synthesis [11]. Therefore, to understand the differences between clones and to define their response to the *rol*B gene, it was necessary to examine some physiological parameters in *D. muscipula* plants.

The main goal of this study was to state the effect of *rol*B gene on the (I) lipid peroxidation level, (II) synthesis of proline, and (III) enzymatic and non-enzymatic antioxidant system activity in transformed clones of the Venus flytrap. Moreover, using Fourier transform infrared spectroscopy (FTIR), the lipid and sugar metabolism of transformed plants was evaluated for the first time in the context of genetic transformation. We hypothesised that transformation with the *rol*B oncogene would trigger the complex and pleiotropic effect manifested by changes in redox state, as well as primary and secondary metabolism of examined organisms.

## Results

### Accumulation of malondialdehyde (MDA) and proline

**Fig. 2:**
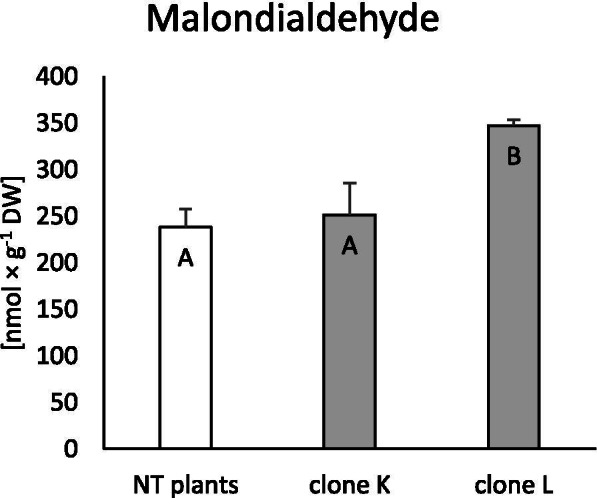
Accumulation of malondialdehyde (MDA) in non-transformed and transformed tissue of *Dionaea muscipula* clones. Different letters indicate significant differences between means at *p* < 0.05; the bar represents the standard deviation

**Fig. 3:**
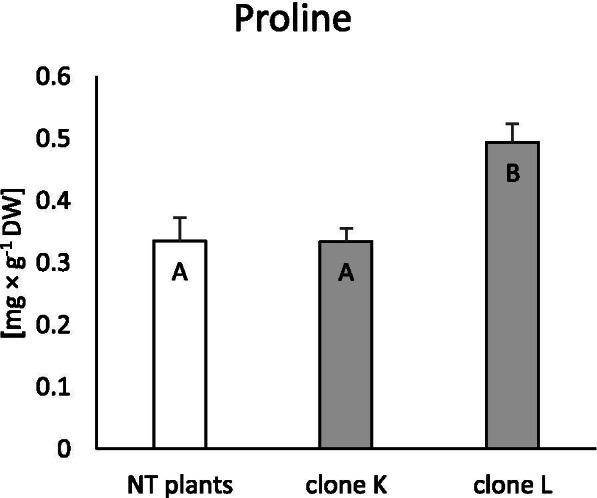
Accumulation of proline in non-transformed and transformed tissue of *Dionaea muscipula* clones. Different letters indicate significant differences between means at *p* < 0.05; the bar represents the standard deviation

Membrane integrity was evaluated as the accumulation of MDA, a product of lipid oxidation ROS. Compared to non-transformed (NT) plants, only clone L accumulated significantly more MDA (46%), while in clone K, the level of MDA did not change (Fig. 2). Similarly, synthesis of the free amino acid proline only increased in clone L (47%; Fig. 3).

### Enzymatic antioxidant system—activity of peroxidase (POD), catalase (CAT), and superoxide dismutase (SOD)

In the presented research, the total activity of three antioxidant enzymes was evaluated. Significantly decreased POD activity was found in transformed clone K tissue, compared to the control. In contrast, CAT was significantly more active (ab. 54%) in clone L. Moreover, the transformation of *D. muscipula* plants led to increased SOD activity in both examined clones in comparison to NT plants (Table 1).

**Table 1:** Activity of antioxidant enzymes: peroxidase (POD), catalase (CAT) and superoxide dismutase (SOD) in non-transformed and transformed tissue of *Dionaea muscipula* clones. Different letters in columns – significant differences between means at *p* < 0.05, SD – standard deviation

	POD [U × g^− 1^ DW ± SD]	CAT [μmol H_2_O_2_ × min^− 1^×g^− 1^ DW ± SD]	SOD [U × g^− 1^ DW ± SD]
NT plants	24.32^B^ ± 3.00	527.35^A^ ± 151.01	111.22^A^ ± 10.25
clone K	20.00^A^ ± 0.83	428.76^A^ ± 88.81	547.50^B^ ± 78.21
clone L	21.85^AB^ ± 0.61	811.56^B^ ± 99.02	554.62^B^ ± 24.19

#### Non-enzymatic antioxidant system

### Total pool of glutathione (GSH + GSSG), reduced (GSH) and oxidised (GSSG) forms, and proportions of reduced and oxidised form (GSH/GSSG)

**Fig. 4:**
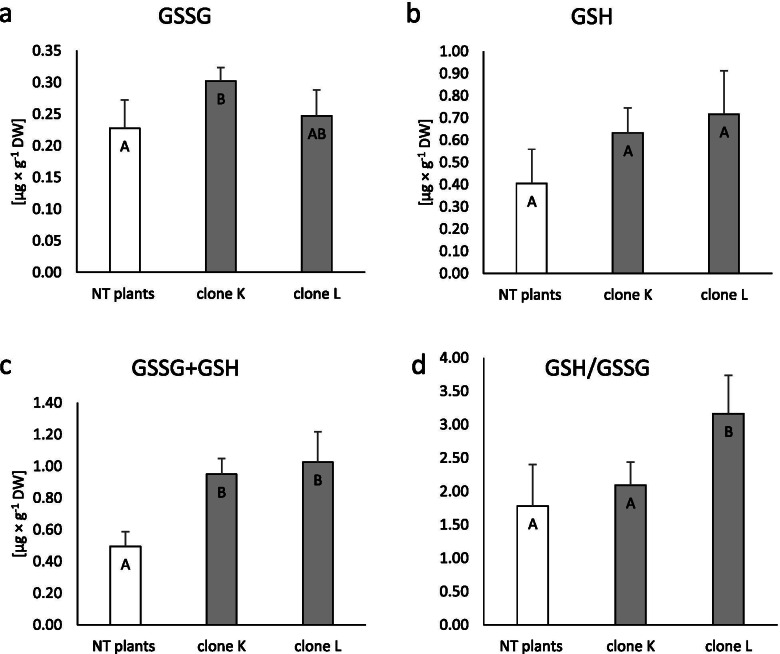
Accumulation of glutathione in non-transformed and transformed tissue of *Dionaea muscipula* clones. **a** Oxidised glutathione (GSSG). **b** Reduced glutathione (GSH). **c** Total glutathione (GSH + GSSG). **d** Glutathione ratio (GSH/GSSG). Different letters indicate significant differences between means at *p* < 0.05; the bar represents the standard deviation

GSH + GSSG increased significantly in both transformed clones compared to NT plants (Fig. 4c), although individual analysis of GSH and GSSG showed that only the GSSG content increased significantly in clone K tissue (33%; Fig. 4a,b). The ratio between the reduced and oxidised forms of glutathione, calculated as the stress indicator, increased significantly in clone L compared to control plants (Fig. 4d).

#### Carotenoid content

**Fig. 5:**
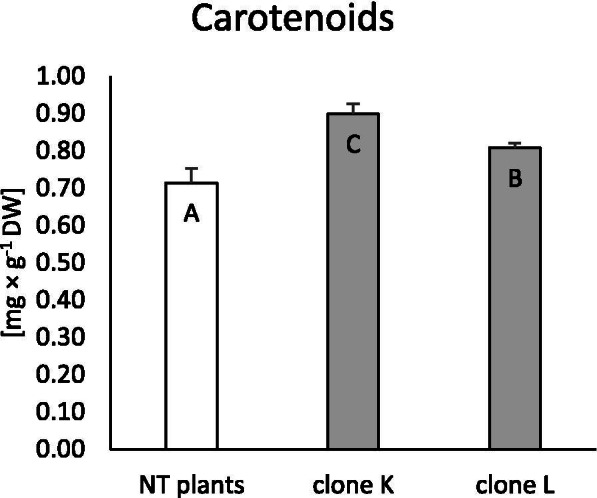
Accumulation of carotenoids in non-transformed and transformed tissue of *Dionaea muscipula* clones. Different letters indicate significant differences between means at *p* < 0.05; the bar represents the standard deviation

One of the important elements of the non-enzymatic antioxidant system in plants is carotenoids. In the present study, estimation of these pigments showed that both transformed clones of the Venus flytrap synthesised significantly more carotenoids than NT plants. Moreover, clone K had a significantly higher carotenoid content (26%) than clone L (Fig. 5).

#### Phenolic compound accumulation

Transformation of *D. muscipula* plants significantly affected phenolic compound synthesis. The HPLC-DAD method was used to determine the content of five phenolic acids—chlorogenic acid, p-coumaric acid, ferulic acid, gallic acid, and protocatechuic acid—and one flavonoid—kaempferol—in extracts from transformed and non-transformed plants (Table 2).

**Table 2:** Accumulation of phenolic compounds in non-transformed and transformed tissue of *Dionaea muscipula* clones. Different letters in lines – significant differences between means at *p* < 0.05, SD – standard deviation

Phenolic compound [mg × 100 g^-1^ DW ± SD]	NT plants	clone K	clone L
gallic acid	31.14^A^ ± 0.48	28.89^A^ ± 1.58	53.73^B^ ± 0.63
protocatechic acid	29.12^B^ ± 0.63	26.18^A^ ± 2.70	24.20^A^ ± 0.57
chlorogenic acid	20.60^A^ ± 0.26	18.83^A^ ± 2.54	28.02^B^ ± 0.48
p-coumaric acid	4.19^B^ ± 1.53	1.09^A^ ± 0.14	8.42^C^ ± 0.28
ferulic acid	16.07^A^ ± 1.32	39.05^B^ ± 0.87	61.49^C^ ± 13.39
kaempferol	58.56^B^ ± 1.65	127.04^C^ ± 2.83	18.30^A^ ± 0.37

The differences were found in the amounts of individual estimated compounds among NT plants and both clones. In NT plants and clone K, the major phenolic compound was kaempferol, except that its accumulation was twice as high in clone tissues (Table 2). In clone L tissues, the main phenolic compounds were gallic acid and ferulic acid (Table 2). In turn, in all examined plants, the minor phenol was p-coumaric acid, although its content differed significantly among tested plants (Table 2). Furthermore, accumulation of protocatechuic acid decreased in both clones in comparison to NT plants, and accumulation of chlorogenic acid increased only in clone L tissue (Table 2).

#### Effect of plant transformation on lipids, sugars, phenolics, and tyrosine

**Fig. 6:**
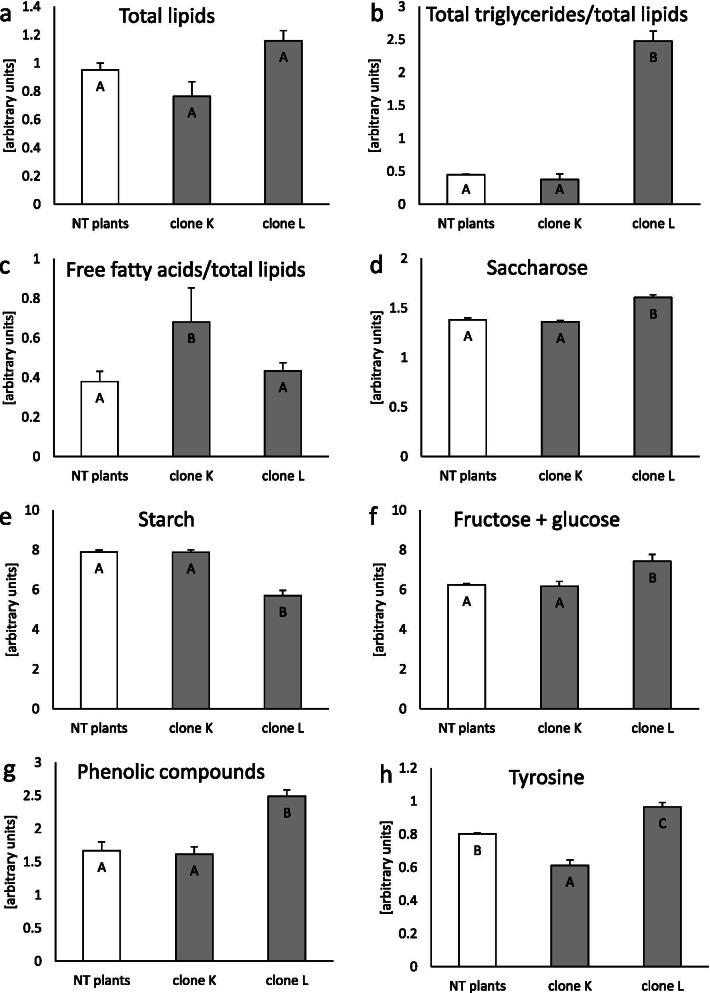
Semi-quantification biocomponents in non-transformed and transformed tissue of *Dionaea muscipula* clones, identified in ATR-FTIR spectra. **a** Total lipids. **b** Total triglycerides/total lipids. **c** Free fatty acids/total lipids. **d** Saccharose. **e** Starch. **f** Fructose + glucose. **g** Phenolic compounds. **h** Tyrosine. Different letters indicate significant differences between means at *p* < 0.05; the bar represents the standard deviation

Spectral lipidomics indicated different changes in the total lipid content (decreased in clone K, increased in clone L) and in the contribution of triacyclglycerols and free fatty acids to the lipidic composition (i.e., increased concentrations were determined for clone L and K; Fig. 6a, b, c). Numerous bands below 1200 cm^− 1^ originated from sugars, and the spectral indicators of soluble and insoluble carbohydrates were selected based on reference FTIR spectra [18–20]. The soluble mono- (fructose and glucose) and disaccharides (saccharose) were overproduced only in clone L, in addition to the decomposition of insoluble starch. The control and clone K contained a similar content of all sugars (Fig. 6d, e, f). All absorbances assigned to the ring modes in the phenyl moieties indicated a significant increase of the phenolic compounds in clone L (showed for the 1611 cm^− 1^ band only, Fig. 6g). In turn, the intensity of the band attributed to the tyrosine ring vibrations dropped down in the spectrum of clone K and increased for clone L compared to the non-transformed plants (Fig. 6h).

## Discussion

*D. muscipula* is a rich source of phenolic compounds, e.g., flavonoids, phenolic acids, and 1,4-naphthoquinone. That is why this plant can be a suitable model for research on plant secondary metabolism [16]. For this reason, medical plant biotechnology uses genetic transformation with *R. rhizogenes* [2]. The *rol* gene family, derived from bacterial T-DNA and transferred to the plant genome, can act as an endogenous elicitor of secondary metabolites, allowing study of the production of valuable plant-derived chemicals in transformed organisms with stable phenotypes and genotypes [3]. Moreover, Veremeichik et al. [21] showed that the effect of *rol* gene action on plant metabolism is long-lasting and repeatable. However, little is known about the physiological response of plants to *rol* oncogene action.

In our previous study, transformation of the Venus flytrap using wild strains of *R. rhizogenes* led to the incorporation of a single copy of the *rol*B gene into plant DNA [11]. Such transformation occurs due to changes in the plant’s growth rate, accumulation of DW, and synthesis of phenolic compounds. Clone K was characterised by a decreased growth rate with simultaneous increased DW content, while clone L grew significantly faster than clone K and non-transformed plants. Additionally, clone L synthesised an increased amount of various phenolic compounds, which positively affected the antibacterial properties of this clone [11].

The subject of this study was to define how the *rol*B gene affects *D. muscipula* physiology at the level of oxidative stress response, primary and secondary metabolism, in two clones, K and L. Tusevki et al. [8] hypothesised that transformation of *Hypericum perforatum* L. plants with wild strains of *R. rhizogenes* modifies cell redox status and consequently leads to an oxidative stress response. Furthermore, it was demonstrated that the *rol*B oncogene can suppress ROS production and activate secondary metabolism in transformed cells, while the mechanism of such an event is still poorly understood [12]. In the present research, MDA content was estimated in transformed plants as the oxidative stress marker. It is well known that an imbalance in ROS production can lead to cell membrane damage, where MDA is a product of lipid peroxidation [22]. Clone L had increased production of MDA, while clone K produced MDA on the same level as non-transformed plants (control). According to Franklin et al. [23], MDA synthesis decreased in cells of *H. perforatum* during treatment with *Agrobacterium tumefaciens*. In contrast, MDA content was increased in *H. perforatum* hairy root lines obtained after transformation with *R. rhizogenes*, compared to non-transformed plants [8]. The upregulation of MDA synthesis in transformed plants may be connected to fast growth in transformed organs and quick development, regardless of the increased activity of antioxidants [8]. Increased mitochondrial respiration during enhanced teratoma development may be a potential donor of huge amounts of ROS, which may lead to lipid-membrane peroxidation [24].

One of the most important elements of plant physiology is the amino acid proline, which is involved in a number of developmental processes, protein synthesis, and stress-related responses, acting as an osmolyte and non-enzymatic antioxidant [25]. Genetic transformation of wheat plants with upregulated proline synthesis and increased tolerance to salt stress was reported by Sawahel and Hassan [26]. Moreover, proline biosynthesis coupled to the pentose phosphate pathway can stimulate the production of phenolics [27]. Tovato et al. [25] discussed the role of proline in the formation of hairy root phenotypes in plants containing the *rol*D gene after *R. rhizogenes* infection. They postulated that apart from auxins, proline can play an important role in hairy root development and elongation. Transformation of *D. muscipula* with the *rol*B gene led to increased production of proline in clone L, while such transformation did not affect the creation of the hairy root phenotype. Accelerated proline synthesis in clone L could be the consequence of the fast growth and demand for primary metabolites, such as proteins in fast teratoma (transformed shoots) development [25]. However, it may be the result of increased oxidative stress in plant tissues, acting as a protectant metabolite in antioxidant defence mechanisms [28].

Oncogenes from *R. rhizogenes* make plant cells more resistant to environmental stress and can inhibit ROS accumulation [7]. The *rol*B gene can greatly activate secondary metabolism, including the activity of antioxidant proteins CAT, POD, or SOD [6, 21]. These enzymatic proteins neutralise ROS upon biotic or abiotic stress. Nevertheless, the mechanism by which the *rol*B gene stimulates the protein antioxidant system remains unknown [8]. Veremeichik et al. [12] showed that the *rol*B gene regulates the expression of NADPH oxidase in *Arabidopsis thaliana* and *Rubia cordifolia* transformed calli, while activity of this enzyme is one of the main sources of ROS production during plant-pathogen interactions. Moreover, it has been postulated that *rol* genes induce the reprogramming of transformed plant cells and provoke pleiotropic effects on primary and secondary metabolism, including enzymatic and non-enzymatic antioxidant systems [29]. Increased CAT, SOD, and ascorbic peroxidase (APX) activity was reported in transformed hairy roots of *H. perforatum* in comparison to non-transformed plants [8]. Kohsari et al. [30] also showed increased SOD and POD activity in hairy roots of *Trigonella foenum-graeceum* and *Trigonella monantha* compared to other organs of these species. Moreover, in *R. cordifolia* callus tissue transformed with the *rol*B gene, Shkryl et al. [31] demonstrated an increase in total POD activity and enhanced abundance in the transcripts of major POD genes. Interestingly, our study on transformed clones of *D. muscipula* demonstrated decreased POD activity in the tissue of clone K, with no changes in POD activity in clone L, in comparison to control plants. Furthermore, CAT activity increased in clone L, and SOD activity was enhanced in both examined clones. These findings may be interpreted as the consequence of increased oxidative stress levels in transformed *D. muscipula* clones. SOD is the first defence against ROS in plants, converting superoxide radicals to hydrogen peroxide [32]. Probably, oxidative stress in clone K does not require the increased action of POD and CAT, being on the same level as non-transformed plants. CAT catalyses the decomposition of hydrogen peroxide and only works actively in high hydrogen peroxide concentrations, while lower doses of hydrogen peroxide may be eliminated by POD [29]. The lack of increased POD activity in clone L could be compensation for SOD and CAT activity [8]. However, plant cells try to resist ROS by adjusting the available antioxidant machinery at the right place and time [4].

Another important element of the cellular redox state is a pool of GSSG and GSH, while the GSH/GSSG ratio is known as the oxidative stress indicator [33]. The results showed that clone K is characterised by an increased pool of GSSG and GSH + GSSG. In turn, clone L has an increased GSH/GSSG ratio and GSH + GSSG. Bulgakov et al. [4] reported that *R. cordifolia* plants transformed with the *rol*B gene had a slightly increased total pool of glutathione and GSH/GSSG ratio. Moreover, *A. thaliana*, after transformation, had an enhanced GSH/GSSG and increased GSH accumulation. Our results confirmed that *rol*B oncogene expression may affect redox homeostasis in plant cells, which results not only in the activity of enzymatic antioxidants but also in the production of non-enzymatic antioxidants.

To support this hypothesis, the carotenoid and phenolic acid content of Venus flytrap teratomas was examined, which together with glutathione are an important element of the non-enzymatic antioxidant system in plants [34]. Both examined clones had an increased carotenoid content compared to the control. Moreover, clone K accumulated more carotenoids than clone L. Furthermore, transformation with the *rol*B oncogene led to changes in phenolic acid synthesis. Clone L synthesised more gallic, chlorogenic, and p-coumaric acid than clone K and control plants. Ferulic acid levels were increased in both clones. Clone K had the highest kaempferol synthesis, while protocatechuic acid synthesis decreased in both examined clones. These results are in agreement with findings by Makowski et al. [11], where clone L had an increased content of total phenolics and selected phenolic derivatives, while clone K accumulated phenolic compounds at the same level as control plants, or lower. Furthermore, other authors reported that plant transformation with wild strains of *R. rhizogenes* can lead to increased phenolic acid production [3, 35, 36]. This phenomenon may result from the fact that expression of the *rol*B gene enhances the activity of phenylalanine ammonia-lyase (PAL) [8]. PAL is a crucial protein in phenolic compound production, catalysing the reaction of *trans*-cinnamic acid synthesis in plant cells. *R. rhizogenes*-mediated transformation enhanced *PAL* expression and stimulated phenylpropanoid metabolism in *H. perforatum* [23]. Simultaneously enhanced growth and increased secondary compound production in response to transformation probably may occurred because of unlimited nutrition resources in vitro conditions. In situations when resources are limited and plants are under stress, a reduction in growth and development may be observed [27].

In the present study, for the first time, the ATR-FTIR technique was combined to estimate some metabolomic parameters in transformed clones of *D. muscipula*. This technique measured lipid, sugar, and protein metabolism changes, demonstrating how the *rol*B oncogene influences plants’ physiology. Lipids are the major components of membranes and have a crucial role in stress signalling in plants [37]. Studying lipid membrane compatibility and composition may show the physiological status of plant cells under stress conditions [38]. Our results showed that plant transformation did not affect the total lipid concentration. Nevertheless, clone L was characterised by an increased triglyceride content in the total lipid content. Furthermore, clone K’s response to *rol*B gene action was manifested by an increased level of free fatty acids. Such changes in lipid compositions following plant transformation may be the result of increased ROS action and lipid oxidation or/and changes in membrane permeability, which enables cell-cell communication and transport [39]. Walley et al. [40] reported that modulation of fatty acid metabolism is one of the elements in complex plants’ response during the plant-pathogen interaction. Additionally, infection and transformation of plants with *R. rhizogenes* or *Rhizobium tumefaciens* (former: *A. tumefaciens*) affect sugar transport and metabolism, while the precise mechanism of action for *rol* oncogenes in sugar metabolism remains unclear [5]. Potato transformation with the *rol*C gene from *R. rhizogenes* changed the accumulation pattern of starch, glucose, and dry matter [41]. Grishchenko et al. [42] postulated that *rol* genes are involved in sugar metabolism through the regulation of enzyme activity, including by glycanases and esterases. These enzymes contribute to the structure of polysaccharides in plant cells. Furthermore, Grishchenko et al. [42] discussed that rather than the *rol*C gene, *rol*B expression can modulate the structure of saccharides in cell walls or plastids. Our examinations of transformed *D. muscipula* plants showed that sugar metabolism was affected by the *rol*B gene only in clone L. Transformation of this clone led to decreased accumulation of starch with simultaneously increased levels of soluble sugars: saccharose and the sum of fructose and glucose. This may be a consequence of faster primary metabolism in clone L. In our previous findings, this clone was characterised by an enhanced growth rate [11]. In such a scenario, the plant needs simple sugars for primary processes and development [43]. It can also be postulated that increased accumulation of simple sugars is connected with oxidative stress in clone L teratomas [44]. In addition to the role in plant growth and development, sugars play a crucial role in signalling cross-talk during the response to environmental stress [44].

Analysis of FTIR spectra also confirmed our findings about phenolic compound accumulation in clones K and L. In our previous paper, teratomas of clone L accumulated an increased quantity of phenolic compounds [11]. In this article, the same trend was observed for phenolic acids evaluated with HPLC. Using a very sensitive technique, FTIR, it was demonstrated that clone L accumulated more phenolic compounds than the control and clone K.

In the context of plant transformation, the synthesis of tyrosine is an important element. In contrast, this aromatic amino acid is the product of primary metabolism. It is used as the precursor for phenolic compound synthesis in the first step of the phenylpropanoid pathway [28]. However, it is postulated that the *rol*B oncogene encodes proteins with tyrosine phosphatase activity, which is crucial in the oncogenesis process [9]. Clone L had an increased accumulation of tyrosine, while the level of this amino acid decreased in clone K. This corresponds with the hypothesis of Bulgakov [9] that the tyrosine phosphatase function of the *rol*B gene enhances secondary metabolism, while dephosphorylation of tyrosine in proteins is an element of the pleiotropic effect of the *rol*B oncogene in plant cells.

## Conclusions

Transformed plants of *D. muscipula* are a new source of knowledge about the physiology of transgenic organisms. Examination of two clones, K and L, showed differences in the response of these plants to transformation events, although both were transformed with a single copy of the *rol*B gene. The pleiotropic effect of the *rol*B gene in transformed plants may be manifested by the regulation of primary and secondary metabolism. The example of clone L showed that transformation with *R. rhizogenes* may lead to enhanced primary and secondary metabolism, as well as promotion of the antioxidant system. Analysis of clone K showed that incorporation of the *rol*B gene in plant genomic DNA does not always cause significant physiological changes. Understanding the mechanisms involved in plant responses to transformation with the *rol*B gene needs further research.

## Methods

### Plant material

In this study, the plant materials were two transformed clones (teratomas) of *D. muscipula*. Plants were transformed with two wild *R. rhizogenes* strains: LBA 9402 (clone K) and ATCC 15834 (clone L; Fig. 1). Plant transformation and selection process, as well as molecular confirmation of transformation, were described by Makowski et al. [11].

For this research, non-transformed plants (NT plants) and transformed clones were cultivated using in vitro conditions [17]. Briefly, plants were grown in liquid ½ strength Murashige and Skoog medium (½ MS) [45] with no growth regulators, 3% sucrose, and pH = 5.5 (adjusted prior to autoclaving), with rotary shaking (130 rpm). Plants were cultivated at a temperature of 23 ± 1 °C, in fluorescence light at 80 × mol × m^2^ × s^1^ photosynthetic photon flux density (PPFD) and a photoperiod of 16 h/8 h light/dark cycle. NT plants and transformed teratomas of both clones were cultivated in 10 biological repetitions. Sixty-day-old tissue cultures were harvested, freeze-dried for 72 h, and homogenised for further analysis.

### MDA content estimation

MDA levels were estimated according to Dhindsa et al. [46], with modifications [47]. Plant tissue was extracted in 1 ml of 0.1% trichloroacetic acid (TCA) solution at 4 °C and centrifuged for 15 min at 25155×g. Subsequently, 0.2 ml of the obtained supernatant was mixed with 0.8 ml of 20% TCA and 0.5% thiobarbituric acid (TBA). Samples were incubated at 95 °C for 30 min and centrifuged for 10 min at 25155×g. The absorbance of mixtures was measured at 532 and 600 nm. Each spectrophotometric analysis in this study was done using a double beam spectrophotometer U-2900 (Hitachi High-Technologies Corporation, Tokyo, Japan). The content of MDA was calculated using the absorbance coefficient for MDA (ε = 155 mM cm^− 1^) after reduction of the value at 532 nm by the correction value at 600 nm. The results were expressed as nM MDA per 1 g of DW tissue.

### Proline content

Accumulation of proline in plant tissue was measured according to Bates et al. [48], with modifications [49]. Dry plant tissue was homogenised in 1 mL 3% aqueous solution of sulfosalicylic acid at 4 °C. Extracts were centrifuged for 15 min, and 0.5 mL was mixed with 0.5 ml acid ninhydrin and 0.5 ml glacial acetic acid. Samples were incubated for 1 h at 100 °C, and the reaction was stopped on ice. Toluene (1 ml) was used to extract the reaction mixture. Absorbance was measured at 520 nm, and the proline concentration was determined from a calibration curve. Calibrations were made with L-proline (Sigma-Aldrich Chemie, GmBH, Steinheim, Germany) as the standard. The results were expressed as mg of proline per 1 g of DW tissue.

### Antioxidant enzyme activity

To estimate the CAT, POD, and SOD activity, native proteins were extracted from dry plant material (20 mg) using 2 mL potassium phosphate buffer (0.05 M, pH = 7.00) at 4 °C. The samples were centrifuged for 15 min at 25155×g (4 °C). The obtained supernatant was collected for protein content estimation and analysis of enzyme activity. All measurements were performed using a double beam spectrophotometer U-2900 (Hitachi High-Technologies Corporation, Tokyo, Japan).

The protein concentration in the extract was determined using the Bradford reagent and bovine serum albumin (BSA) as a standard [50]. CAT activity was determined using the method described by Aebi [51] with modifications by Tokarz et al. [52]. The supernatant (0.2 mL) was mixed with 1.8 mL phosphate buffer (pH 7.0) and 1 mL H_2_O_2_ solution in phosphate buffer. The absorbance of H_2_O_2_ decomposed by the enzyme was measured at 240 nm for 4 min in 1-min intervals. The results were presented as the amount of enzyme that decomposed 1 μmol H_2_O_2_ in 1 min.

The POD activity was determined using the spectrophotometric method by Lück [53], with modifications by Tokarz et al. [52]. This method was based on the reaction of p-phenyldiamine oxidation to phenazine by the tested enzyme. Phosphate buffer (1.5 mL; pH 6.2), supernatant (0.5 mL), and 1% p-phenyldiamine solution (0.1 mL) was mixed with 0.1 mL 0.1% H_2_O_2_. The absorbance was measured at 485 nm (0.1 rise of absorbance correspond to one unit of POD activity).

SOD activity was measured according to Hwang et al. [54], with modifications [34]. The enzyme extract was mixed with methionine, nitro blue tetrazolium, and riboflavin. The mixture was incubated in light (two 18 W fluorescence lamps). Absorbance was measured at 560 nm after 5 and 10 min. A similar mixture without the enzyme was prepared as a control, in which the reaction efficiency reached 100%. One unit of enzyme activity was defined as 50% inhibition of the reaction.

### Non-enzymatic antioxidants

#### Carotenoid accumulation

The dried sample (20 mg) was extracted three times in 1 mL 80% acetone with the addition of MgCl_2_ to discolour plant tissue at 4 °C [55]. The samples were centrifuged for 15 min at 25155×g (4 °C). The absorbance of the diluted supernatant was measured at 470 nm using a double beam spectrophotometer U-2900 (Hitachi High-Technologies Corporation, Tokyo, Japan). The carotenoid content was calculated according to Wellburn [56].

#### Reduced and oxidised glutathione

The glutathione pool was measured according to Queval and Noctor [57], where 5,5-dithiobis(2-nitro-benzoic acid) (DTNB) is glutathione reductase (GR)-dependent reduced. DW tissue (20 mg) was extracted at 4 °C using 1 mL 0.2 N HCl. The samples were centrifuged at 25255×*g* for 10 min at 4 °C. The obtained supernatant (0.5 mL) was neutralised with 0.5 M NaOH in the presence of 50 μL 0.2 M NaH_2_PO_4_ (pH 5.6) to reach a final pH between 5 and 6. The method allowed the measurement of the total glutathione pool (reduced plus oxidised form: GSSG+GSH) and, after pre-treatment of the extract aliquots with 2-vinylpyridine (VPD), only GSSG was measured. To measure GSSG+GSH, aliquots of 30 μL neutralised extracts were added to 300 μL 0.2 M NaH_2_PO_4_ (pH 7.5), 30 μL 10 mM EDTA, 30 μL 10 mM NADPH, 30 μL 12 mM DTNB, and 180 μL distilled water. The reaction was started by the addition of 30 μL GR (20 U mL^− 1^), and the increase in the absorbance at 412 nm was monitored for 2 min. The GSSG fraction was measured using the same routine after incubation of 200 μL neutralised extract with 3 μL VPD for 30 min at room temperature to complex GSH. Calculations were made on the basis of standard curves plotted simultaneously for GSH and GSSG. The GSH/GSSG ratio was also calculated.

#### Phenolic compound estimation using DAD-HPLC

The phenolic compounds were estimated in methanolic extracts prepared from 200 mg DW tissue in 2.5 mL HPLC-grade methanol using sonication (two times for 30 min at 25 ± 2 °C) (Polsonic). Samples were centrifuged (25,255×*g* for 15 min at 4 °C). The obtained supernatant was filtered through syringe filters (0.22 μm Millex®GP, Millipore, Merck, Darmstadt, Germany) for analysis with high pressure liquid chromatography with a diode array detector (DAD-HPLC).

The quantitative analyses of phenolic compounds in the extracts were done by a validated method, using an apparatus from Merck-Hitachi (LaChrom Elite) with a DAD L-2455 detector and a Purospher RP-18 (250 × 4 mm; 5 μm, Merck, Germany) column [58, 59]. The flow rate was 1 mL × min^− 1^, and temperature was set at 25 °C; the injection volume was 10 μL. The detection wavelength was set at 254 nm. The mobile phase consisted of A—methanol, 0.5% acetic acid 1:4 and B—methanol (v/v). The gradient program was as follows: 0–20 min, 0% B, 20–35 min, 0–20% B, 35–45 min, 20–30% B, 45–55 min, 30–40% B, 55–60 min, 40–50% B, 60–65 min, 50–75% B, and 65–70 min, 75–100% B, with a hold time of 15 min. Identification was performed by comparison to retention times and UV spectra of standards (chlorogenic acid, p-coumaric acid, ferulic acid, gallic acid, protocatechuic acid, and kaempferol acquired from Sigma-Aldrich Co., Germany). The quantification was performed based on the calibration curves method. Samples were prepared and analysed in five replications. The results were expressed in mg × 100 g^− 1^ DW ± SD.

#### ATR-FTIR measurements

Various plant organics absorb the mid-infrared light, giving a molecular fingerprint to the chemical composition when examined in the 4000–900 cm^− 1^ region. In our study label-free and rapid FTIR spectroscopy with attenuated total reflection mode (ATR) was used to determine changes in plant tissue composition after transformation with wild *R. rhizogenes* bacteria. Analysis revealed the presence of various characteristic functional groups originating from lipids, phenolic compounds, and a plethora of mono- and polysaccharides (see Additional file 1). Amide I and II bands of proteins were absent. There was an alternation of intensities in IR bands assigned to lipids (stretches of the CH_2_ groups; 2850 cm^− 1^), triglycerides (stretches of the ester C=O groups; 1735 cm^− 1^), fatty acids (stretches of the acidic C=O groups 1718 cm^− 1^), sugars (stretches and deformations of the C-C and C-O groups; saccharose—866 cm^− 1^; starch—1154/1076 cm^− 1^; fructose and glucose—1104/1020 cm^− 1^), phenolic compounds (stretches of the C=C groups; 1611 cm^− 1^), and tyrosine residues (stretches of the C=C groups 1511 cm^− 1^). Their values are the estimate of the content of molecules [18, 19].

Ground, freeze-dried leaves and stems were deposited on an ATR crystal. ATR-FTIR spectra were recorded with a Bruker Alpha FTIR spectrometer with a single-bounce diamond ATR crystal. For each sample, at least three spectra were acquired with a spectral resolution of 4 cm^− 1^ in the region of 4000 to 600 cm^− 1^ by co-adding 64 scans. Spectra pre-processing and analysis were performed using OPUS software (Bruker Optics, Bullerica, MA, USA, Version 7.2.139.1294). First, the extended ATR correction was applied as implemented in the software. After vector normalisation in the region of 3700–600 cm^− 1^, the second derivative IR spectra were calculated with 9 smoothing points according to a Savitzky-Golay protocol. Second derivative/absorption spectra were used for the calculation of the integral intensity of various bands. For this purpose, a linear baseline was drawn through the peak edges, and the spectrum below this line was integrated over the wavenumber range of the band. For the comparison of spectral differences between studied groups, spectra from each measurement were averaged within the sample.

#### Statistical analyses

Statistical analyses were performed using STATISTICA 12.0 (StatSoft Inc., Tulsa, OK, USA). The results were subjected to one-way analysis of variance (ANOVA), and the significance of differences between the arithmetical means was determined by Tukey’s post hoc test at *p* ≤ 0.05.

## Supplementary Information

**Additional file 1: **Averaged ATR-FTIR spectra (**a**) and their second derivatives (**b**) (±SD) of non-transformed and transformed tissue of *Dionaea muscipula* clones.

## Data Availability

The datasets used and/or analysed during the current study are available from the corresponding author upon reasonable request.

## Abbreviations

ROS: Reactive oxygen species
T-DNA: Transfer DNA
FTIR: Fourier transform infrared spectroscopy
DW: Dry weight
MDA: Malondialdehyde
POD: Peroxidase
CAT: Catalase
SOD: Superoxide dismutase
GSH: Reduced form of glutathione
GSSG: Oxidised form of glutathione
NT plants: Non-transformed plants
APX: Ascorbic peroxidase
PAL: Phenylalanine ammonia lyase
TCA: Trichloroacetic acid
TBA: Thiobarbituric acid
DTNB: 5,5-dithiobis(2-nitro-benzoic acid)
BSA: Bovine serum albumin
PPFD: Photosynthetic photon flux density
