# Supplementary material for: Response of physiological parameters in Dionaea muscipula J. Ellis teratomas transformed with rolB oncogene
Source: BMC Plant Biol. 2021 Nov 29;21:564. doi: 10.1186/s12870-021-03320-y (PMC8628454; doi:10.1186/s12870-021-03320-y)
Supplement: Supplementary file 1 [file 12870_2021_3320_MOESM1_ESM.docx]

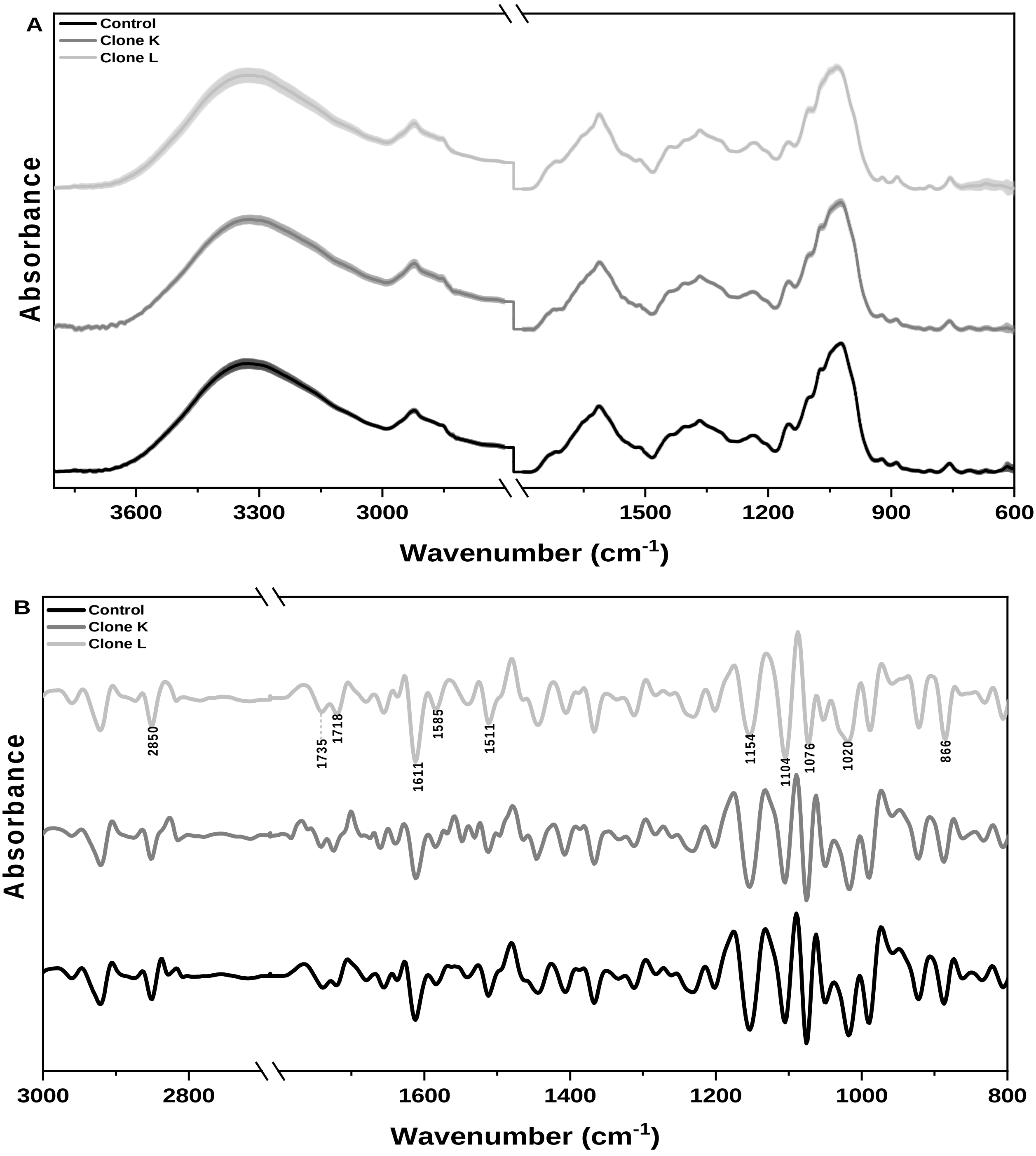
A
Control
Clone K
Clone L
Absorbance
3600
3300
3000
1500
1200
900
600
Wavenumber (cm-1)
B
Control
Clone K
Clone L
Absorbance
2850
1735
1718
1611
1585
1511
1154
1104
1076
1020
866
3000
2800
1600
1400
1200
1000
800
Wavenumber (cm-1)
